# A Diverse Viral Community from Predatory Wasps in Their Native and Invaded Range, with a New Virus Infectious to Honey Bees

**DOI:** 10.3390/v13081431

**Published:** 2021-07-23

**Authors:** Emily J. Remnant, James W. Baty, Mariana Bulgarella, Jana Dobelmann, Oliver Quinn, Monica A. M. Gruber, Philip J. Lester

**Affiliations:** 1 Behaviour, Ecology and Evolution Laboratory, School of Life and Environmental Sciences, Science Road, University of Sydney, Sydney, NSW 2006, Australia; 2 School of Biological Sciences, Victoria University of Wellington, P.O. Box 600, Wellington 6140, New Zealand; james.baty@vuw.ac.nz (J.W.B.); Mariana.Bulgarella@vuw.ac.nz (M.B.); jana.dobelmann@uni-ulm.de (J.D.); quinnol@hotmail.com (O.Q.); monica.gruber@vuw.ac.nz (M.A.M.G.); phil.lester@vuw.ac.nz (P.J.L.); 3 Institute of Evolutionary Ecology and Conservation Genomics, Department of Biology, University of Ulm, Albert-Einstein-Allee 11, 89081 Ulm, Germany; 4 Bacteriology and Aquatic Animal Diseases, Ministry for Primary Industries, P.O. Box 2526, Wellington 6140, New Zealand

**Keywords:** multi-host pathogen, spillover, wasp virus, honey bee virus, virus discovery

## Abstract

Wasps of the genus *Vespula* are social insects that have become major pests and predators in their introduced range. Viruses present in these wasps have been studied in the context of spillover from honey bees, yet we lack an understanding of the endogenous virome of wasps as potential reservoirs of novel emerging infectious diseases. We describe the characterization of 68 novel and nine previously identified virus sequences found in transcriptomes of *Vespula vulgaris* in colonies sampled from their native range (Belgium) and an invasive range (New Zealand). Many viruses present in the samples were from the Picorna-like virus family (38%). We identified one Luteo-like virus, Vespula vulgaris Luteo-like virus 1, present in the three life stages examined in all colonies from both locations, suggesting this virus is a highly prevalent and persistent infection in wasp colonies. Additionally, we identified a novel Iflavirus with similarity to a recently identified Moku virus, a known wasp and honey bee pathogen. Experimental infection of honey bees with this novel Vespula vulgaris Moku-like virus resulted in an active infection. The high viral diversity present in these invasive wasps is a likely indication that their polyphagous diet is a rich source of viral infections.

## 1. Introduction

Insects have been shown to harbor a remarkable viral diversity with a high abundance of novel virus families [1,2]. This viral diversity has important implications for our understanding of infectious diseases in economically, ecologically, and epidemiologically important insects. The viromes of social insects are becoming increasingly well characterized with a recent focus on identification of novel viruses in honey bees and other pollinators [3,4,5,6,7]. Many of these viruses infect a wide variety of hosts, with a high risk of virus spillover and therefore the potential for emerging infectious diseases among organisms in shared ecological space [8,9].

Pollinator networks exemplify this scenario, with a wide range of interacting arthropods occurring in sympatry, providing plentiful opportunities for interspecies virus transmission [10,11]. Although originally classified as honey bee viruses, many bee-associated viruses are multi-host pathogens [11,12,13]. A prominent example is deformed wing virus (DWV), a Picornavirus from the Iflaviridae family. DWV is the cause of a recent epidemic in honey bees, spurred on by the global spread of the mite *Varroa destructor* as a vector [14,15]. The arrival and spread of *Varroa* into new environments, such as Hawai’i [14] and New Zealand [16], has caused a dramatic shift in the honey bee viral landscape. Virus transmission between honey bees and sympatric alternate hosts can be direct or indirect and include physical contact between species or contact on shared resources such as contaminated flowers and pollen, or via robbing of stored food from colonies or predation of infected individuals [11,17,18]. The spillover of honey bee-associated viruses is not only evident in wild bee pollinators, but also in other organisms that occur in sympatry, like ants, cockroaches, and wasps [18,19,20,21,22,23].

Wasps from the genus *Vespula* have a native Holarctic distribution [24]. They have successfully established across the globe, including more recently in South America, South Africa, Australia, and New Zealand [25,26]. *Vespula* are voracious, opportunistic omnivores, preying on a diverse range of insects and scavenging invertebrate and vertebrate carcasses, supplementing these protein-rich sources with energy-rich honeydew and nectar [25,27]. Honey bee colonies are common targets for wasps, which frequently prey and scavenge on adult bees, rob hives of honey, and forage on shared floral resources [22,28,29].

*Vespula* wasps are known to harbor a diverse microbial fauna, experiencing many of the same pathogens as honey bees [18,30,31]. Multiple viruses are able to replicate in *Vespula* wasps and can result in detrimental fitness effects, suggesting that viruses can be significant parasites [18,20,32,33]. A high prevalence of bee-associated viruses have been observed in *V. vulgaris* in the USA [11], New Zealand [34], and *V. pensylvanica* in Hawai’i [19], with viral sequence similarities and loads likely to be associated with proximity to local honey bees.

While the majority of viruses identified in wasps have been previously described from honey bees, a novel Iflavirus, Moku virus, was recently isolated in the wasp *Vespula pensylvanica* [5]. Moku virus has also been detected in neighboring honey bees and *Varroa* and has since been identified in hornets (*Vespa velutina* [35,36]) and other wasp species, with frequent detections in *V. vulgaris* [37,38]. Moku virus loads are typically much higher in *V. pensylvanica* compared to co-located invertebrates, suggesting a possible host preference for wasps [19]. The phenotypic impacts of Moku virus infection are largely unknown, however higher Moku viral loads have been associated with reduced colony longevity in *V. pensylvanica* [33].

Here, we investigate the viral diversity of *V. vulgaris* using metatranscriptomics. We examine multiple colonies from the native range (Belgium) and from an invasive population in New Zealand. We additionally survey juvenile (larval), adult, and newly emerged reproductive stages (gynes) from each colony to compare virus abundance and prevalence across a range of different life stages. We report the identification of 68 novel virus sequences from a range of viral families, including one novel Luteo-like virus present in all colonies and life stages, and a novel Picorna-like virus similar to the previously identified Moku virus, a known multi-host pathogen identified in Vespids. We examine the host-specificity of this novel Moku-like virus in honey bees to assess the potential for viral spillover in our most important commercial pollinator.

## 2. Materials and Methods

### 2.1. Vespula vulgaris Collections, RNA Extractions and RNA Sequencing

Nests were sampled from three separate sites within the native (Belgium) and invaded (New Zealand) ranges (Table S1, [39]). In Belgium, samples were taken in the area surrounding the city of Leuven. Within Leuven, we were alerted to the presence of nests by city residents discovering mature wasp nests on their properties. In New Zealand, sampling sites were in the Nelson/Tasman district, within native forest ecosystems. All nests were sampled in the autumn season of both ranges in 2014, to allow sampling of three life stages: larvae, workers, and gynes (newly emerged queens), for a total of 18 samples; nine from each range. Five individuals of each life stage per sample were cut up and placed in RNAlater (Sigma-Aldrich, St. Louis, MO, USA) in nuclease-free vials and stored at −80 °C prior to extraction.

Total RNA was extracted using the PureLink RNA Mini Kit (ThermoFisher, Waltham, MA, USA). RNA quantity and integrity was assessed on an Agilent 2100 Bioanalyzer using the RNA 6000 Nano Chip kit. Total RNA transcriptome libraries were made using the TruSeq RNAseq Sample Prep kit (Illumina, San Diego, CA, USA) according to the manufacturer’s instructions. Libraries were sequenced on the Illumina HiSeq platform (2 × 100 bp paired-end) by New Zealand Genomics Limited.

### 2.2. Identification and Phylogenetic Analysis of Novel Virus Sequences

RNA sequencing reads were assembled de novo using Trinity [40]. Assembled contigs were compared to reference protein sequences of a comprehensive database of previously characterized viruses downloaded from GenBank, NCBI (accessed on 11 December 2018) using BLASTx, with an E value of 1-E5. BLASTn and BLASTx searches to a non-redundant database were then performed with resulting virus-like contigs to remove non-viral hits, such as host contigs that show similarity to viral sequences. All putative viral contigs were checked against the *V. vulgaris* genome [26] to ensure that all sequences were exogenous. To determine if the numbers of virus-like contigs differed between different life stages, we compared the mean rank of the contig number in each of the three life stages examined (larvae, workers, and gynes) with a Kruskal–Wallis test, followed by a pairwise Wilcoxon test with the Benjamini–Hochberg adjustment. All analyses were performed in R [41].

The novel virus genome sequences were imported into Geneious [42], with predicted ORFs and amino acid sequences obtained using the ‘translate’ function. The predicted virus protein sequences were aligned to homologous viral proteins using Muscle [43]. Alignments were viewed in Geneious and manually trimmed to remove large gaps and non-conserved regions. Maximum likelihood phylogenetic trees were inferred using IQ-TREE [44], with substitution models determined by ModelFinder [45] (see figure legends for details pertaining to each individual tree). Branch supports were estimated using ultrafast bootstrap approximation (UFBoot; [46]) and SH-aLRT [47]. Nucleotide alignments of Vespula vulgaris Luteo-like virus variants observed in each of the six nests were performed using Muscle and a phylogenetic tree was generated using maximum likelihood in IQ-TREE as above.

### 2.3. PCR Validation of Novel Viruses

The novel Moku-like virus and Luteo-like virus were validated by PCR and Sanger sequencing using primers designed from the RNA transcriptome data (Table S2). Four individual *Vespula vulgaris* sampled from Belgium were selected for virus screening. RNA and DNA was simultaneously extracted using a CTAB and chloroform-based protocol. Briefly, the samples were homogenized by bead-bashing in microcentrifuge tubes containing 1 mL of GENEzol plant DNA reagent (Geneaid Biotech, New Taipei City, Taiwan) and 5 µL of β-mercaptoethanol (Sigma Aldrich, MI, USA) in a Precellys Evolution homogenizer (Bertin Instruments, Montigny-le-Bretonneux, France).

Reverse transcription (RT) to generate cDNA was conducted using 2 µL qScript XLT cDNA SuperMix (QuantaBio, Beverly, MA, USA) with 1 µg RNA in 10 µL reactions. Aliquots of the cDNA were combined into one mixture for quick PCR screening using primer pairs that amplified relatively short products (106 bp, 143 bp and 279 bp for Moku-like virus, and 372 bp for Luteo-like virus). PCR reactions consisted of 0.4 µM forward and reverse primers, 1 µL of cDNA (0.1 µg), and 12.5 µL MyTaq Red Mix (Bioline, London, UK) made to 25 µL with water. The cycling conditions were as follows: 95 °C for 1 min; 35 cycles of 95 °C for 15 s, 60 °C for 60 s, 72 °C for 20 s; and a hold step at 4 °C. PCR products were resolved by 2% agarose gel electrophoresis and visualized using SYBR Safe DNA gel stain (Invitrogen/ThermoFisher Scientific, Waltham, MA, USA). The PCR screen of the pooled cDNA sample resulted in amplification of both Moku-like virus and Luteo-like virus. The four samples were then tested individually and two were positive for Moku-like virus, whereas all four were positive for Luteo-like virus. No amplification was observed from RNase-treated RNA/DNA samples or from the samples pre-RT, ruling out amplification from genomic DNA.

One of the samples positive for Moku-like virus was selected for PCR and Sanger Sequencing with primer pairs that generated long overlapping products covering the entire assembled Moku-like virus sequence (Table S2). Fresh cDNA was generated from 1 µg RNA using SuperScript IV VILO Master Mix with ezDNase enzyme (Invitrogen/ThermoFisher Scientific, Waltham, MA, USA) in 20 µL reactions. The cDNA was then used for PCR in nine separate reactions using a very high fidelity Taq enzyme (Platinum SuperFi PCR Master Mix, Invitrogen/ThermoFisher, Waltham, MA, USA). Reactions consisted of 1 µL cDNA (50 ng), 0.5 µM forward and reverse primers, 10 µL SuperFi PCR Master Mix made to 20 µL with nuclease-free water. Reaction conditions were as follows: 98 °C for 30 s; 35 cycles of 98 °C for 10 s, 60 °C for 10 s, 72 °C for 45 s; followed by 72 °C for 5 min; and then holding at 4 °C. PCR products were resolved by 1% agarose gel electrophoresis to confirm amplification. Samples were then prepared for Sanger sequencing using ExoSAP-IT PCR Product Cleanup Reagent (Applied Biosystems/ThermoFisher Scientific, Waltham, MA, USA). Sequencing was provided by Massey Genome Service (Palmerston North, North Island, New Zealand). Sequences of the PCR products were then aligned with the assembled Moku-like virus sequences using Geneious [42].

Sanger sequencing of the Luteo-like virus PCR products followed the same approach but with an additional PCR step to obtain sequence from a troublesome region. PCR with the three Luteo-like primer pairs (Table S2) led to Sanger sequences that covered the entire assembled Luteo-like virus, however Luteo-like_2364F and Luteo-like_2846R primers generated a faint 483 bp band in the agarose gel so PCR was repeated to amplify the original PCR product.

We attempted to test for active viral replication in the wasp using strand-specific RT-PCR to detect the negative-sense viral strand (method modified from [48,49]). However, repeated attempts with methodological modifications failed to provide clear results.

### 2.4. Virus Propagation and Screening in Honey Bees

In this first experiment, we sought to determine if Moku-like virus would infect honey bees (*Apis mellifera*). We prepared a ‘working inoculum’ of un-purified Moku-like virus by mixing approximately 0.1 g of a homogenized, Moku-like virus positive *Vespula vulgaris* larva with 1 mL of a 1.75 M sucrose solution [50]. Larvae were collected in Belgium in 2015 and kept at −80 °C. The wasp larva was placed in a microtube with three stainless steel beads and homogenized in two cycles of 15 s each at 6000 rpm. The larvae also tested positive for Luteo-like virus.

Prior to an experiment where we inoculated bees, we screened 10 randomly selected bees from a Victoria University of Wellington hive to confirm that the colony was free of Moku-like and Luteo-like viruses. For the feeding experiment, we collected 29 bees from the hive entrance. Each bee was individually immobilized inside a cut 1000 µL pipette tip with the head protruding [51]. Nineteen bees were fed 10 µL of the Moku-like virus-sugar solution from a pipette tip. At the same time, we also fed 10 additional bees (a control treatment) with 10 µL each of the same sugar water solution without virus. Bees ingested the 10 µL solution readily.

The control and Moku-like virus-fed bees were kept in separate plastic cages with a mesh lid (15 × 14 × 5.5 cm), provided with an impregnated sugar-water cotton ball for food and another cotton ball impregnated with water for drinking. Bees were kept inside an incubator at 24 °C, 63% relative humidity and 10 h/14 h light/dark cycle. Bees were checked twice daily, and the sugar solution and water replenished for the seven days of the experiment. All surviving adults were frozen alive on day 7. Experiments were conducted in a PC2 laboratory.

Seventeen of the 19 bees fed Moku-like virus were alive when the experiment ended (on day 7), and we extracted the RNA of these 17 bees. Within the control group, only seven bees were alive when the experiment finished but all 10 bees in the control group had their RNA extracted. We extracted RNA using the Direct-Zol RNA MiniPrep Plus kit (Zymo Research, Irvine, CA, USA). For the homogenization step, each bee was placed in a 2 mL microtube with two 3.2 mm stainless steel beads and 600 µL of TRIzol reagent (Invitrogen/Thermofisher, Waltham, MA, USA) and homogenized for two cycles of 15 s each at 6000 rpm. The rest of the extraction procedure followed the manufacturer’s protocol. We quantified RNA using a NanoPhotometer (Implen, Munich, Germany). Next, we reverse transcribed RNA to cDNA using Quanta qScript XLT cDNA SuperMix (QuantaBio, Beverly, MA, USA). We used PCR to determine the presence or absence of Moku-like virus and Luteo-like virus in each of the sampled bees, with reaction volumes of 15 µL containing 1 × MyTaq Red Mix (Bioline/Meridian Bioscience, London, UK), 5 pmol of each primer (Moku-like-3218F and Moku-like-3496R; Luteo-like-1324F and Luteo-like-1695R), and ultra-pure water (Invitrogen). We visualized PCR products by 2% agarose gel electrophoresis. We purified positive products with rSap combined with Exo 1 (New England Biolabs, Ipswich, MA, USA). Sequencing was performed on an ABI 3130 × 1 Genetic Analyzer (Applied Biosystems/Thermofisher, Waltham, MA, USA) at Massey Genome Service (Palmerston North, New Zealand). We aligned the sequences using the default alignment algorithm implemented in Geneious [42].

### 2.5. Moku-Like Viral Load in Inoculated Honey Bees

Our next experiment quantified Moku-like viral loads in bees after infection. Forty honey bees were each immobilized inside a pipette tip as described above. Each individual bee in the treatment group (*n* = 20) was fed 10 µL of the described virus-sugar solution from a pipette tip. Control group bees were fed 10 µL each of a sugar water solution without virus (*n* = 20). Each of the 20 bees in each treatment was frozen alive at −80 °C at different time intervals. Five bees in each treatment were frozen on day 0 (six hours after they were offered the solution), 1, 5, and 10. Bees were kept in the conditions described above for the previous experiment.

We extracted RNA and reverse transcribed to cDNA as described previously from three bees per treatment and three bees from the control group per day (days 0, 1, 5, and 10; 24 bees in total). The cDNA was diluted with nuclease-free water to 5 ng/µL and 8 µL/sample loaded into each well of the MicroAmp Fast Optical 96-Well Reaction Plate (Applied Biosystems/ThermoFisher Scientific, Waltham, MA, USA), along with 10 µL PowerUp SYBR Green Master Mix (Applied Biosystems/ThermoFisher Scientific, Waltham, MA, USA), and forward and reverse primers at final concentrations of 300 nM (Moku-like_8664F and 8806R, or NDUFA8F and R, or PROS54F and R, Table S2). A QuantStudio 7 Flex Real-Time PCR System (Applied Biosystems/ThermoFisher Scientific, Waltham, MA, USA) was used to analyze the reaction plates with the following fast cycling conditions: (50 °C, 2 min; 95 °C, 2 min; 40 cycles of 95 °C, 1 s, 60 °C, 30 s). Quantification cycle (Cq) values averaged from two technical replicates were used to calculate Moku-like virus levels relative to the reference genes (NDUFA8 and PROS54 [52]) using the equation (2^(−Cq Moku-like virus))/average of (2^(−Cq NDUFA8)) and (2^(−Cq PROS54)). The final reported expression values are the average of three biological replicates with the standard error of the mean (SEM).

## 3. Results

We examined total RNA transcriptomes from three wasp life-stages (larvae, adult workers, and adult virgin queens (referred to as gynes)), each sampled from six mature nests in autumn; three from the native range of Belgium and three from the invaded range in New Zealand (Table S1). The number of virus-like contigs assembled varied greatly between sample types (H(2) = 13.373, *p* = 0.001248), with larvae showing significantly more assembled viral contigs when considering fragments >200 bp (average number for larvae = 207, workers = 57.5, and gynes = 2.33; larvae vs. workers *p* = 0.0411; larvae vs. gynes *p* = 0.0074; workers vs. gynes *p* = 0.0074; Table S3). When considering only larger contigs (>1 kb), gyne samples had significantly fewer compared to workers and larvae: gyne samples contained between 1–3 viral contigs, whereas worker samples ranged from 5–23 and larvae 8–69 (H(2) = 12.554, *p* = 0.001879; gyne vs. larvae *p* = 0.007; gyne vs. worker *p* = 0.007; larvae vs. worker *p* = 0.146). Smaller virus-like contigs were assembled into larger viral genomes based on overlapping homology where possible.

**Figure 1:**
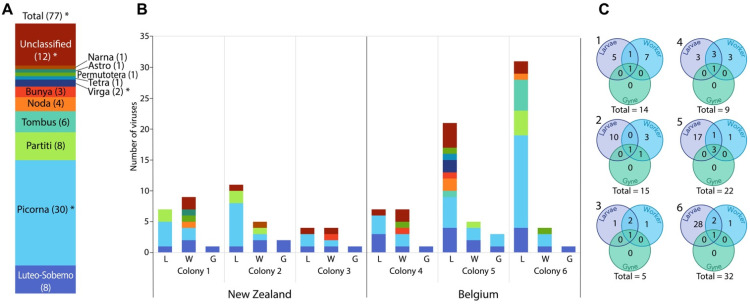
Number of viruses identified in *Vespula vulgaris* colonies from New Zealand and Belgium. (**A**) Total number of viruses identified (77) sorted by viral family. * indicates some previously identified viruses (Total (77) = 68 novel and nine previously identified viruses; of which six previously identified were Picorna, two unclassified, and one Virga). (**B**) Number of viruses identified in each sample from each colony (L = larva, W = worker, G = gyne) sorted by viral family (see also Table S3). (**C**) The overlap of viruses between wasp life-stages within wasp colonies (see also Table S4). Total viruses identified in each colony is indicated.

**Figure 2:**
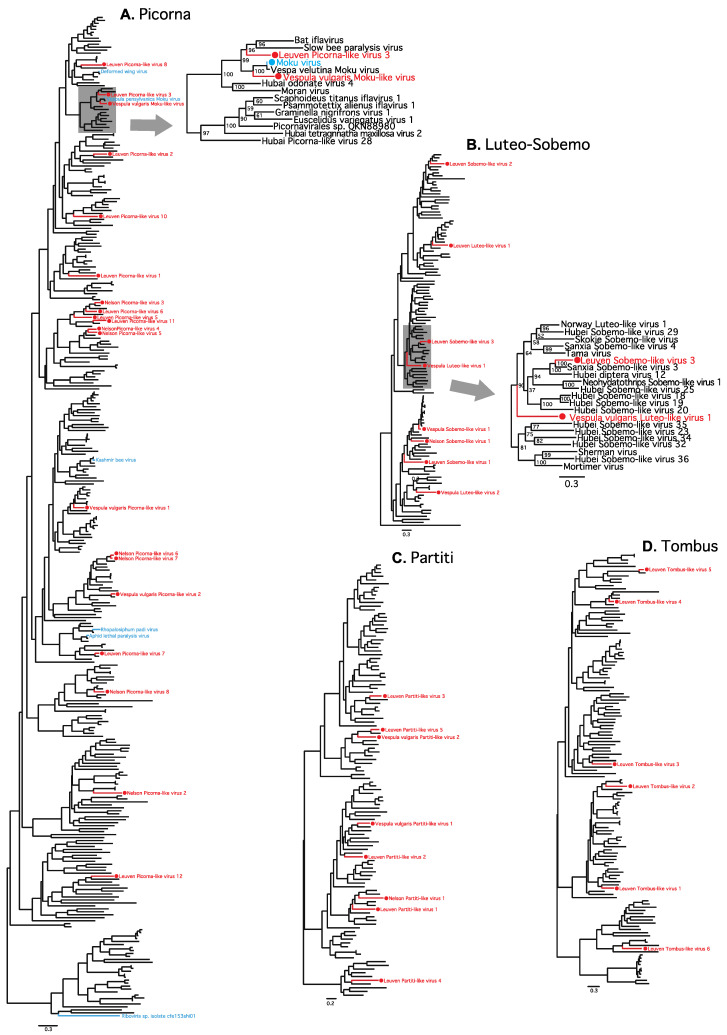
Evolutionary relationships of novel viruses. Shown are maximum likelihood phylogenies from viral families representing the majority of the novel viruses identified in this study (red). (**A**) Picorna. Twenty of the 24 novel Picornaviruses identified (four genomes were incomplete over the RdRp region used for alignment and were thus excluded from the analysis). Six previously identified picornaviruses also present in the samples are indicated (blue). Zoom shows the region of the tree containing the novel Vespula vulgaris Moku-like virus (**B**) Luteo-Sobemo. Eight novel Luteo-Sobemo viruses identified. Zoom shows the region of the tree containing the novel Vespula vulgaris Luteo-like virus 1. (**C**) Partiti. Eight novel Partiti viruses identified. (**D**) Tombus. Six novel Tombus viruses identified. (See Figures S1–S4 for full phylogenetic trees).

We found evidence for 68 novel virus species and nine previously identified virus species across all 18 transcriptomes (Figure 1, Table S4, File S1). Assembled viral genomes ranged in size from 1.4–12 kb (Table S4). Of the 68 novel viruses, 17 were predicted to be partially based on truncated 5′ or 3′ regions of the predicted open reading frames (ORFs), and for nine viruses only a single segment of the typically multi-segmented genome was identified (Table S4). The viral component of each transcriptome comprised approximately 0.04–1% of total RNA (Table S3). Most viruses were present in one sample only (60 virus species; 78%); however, 17 viruses were found in more than one sample; and of these, 13 were found in more than one colony, with four found in both the native and invasive ranges (Table S4). Within colonies, limited consistency was observed between sample types, with only 2–4 viruses shared between at least two life stages (Figure 1C, Table S4). The 68 previously uncharacterized virus sequences exhibited between 23–92% amino acid similarity compared to their most closely related sequence available in NCBI (Table S4, File S1). Most viruses present belonged to the Picorna family (30/77), followed by the Luteo-Sobemo (8), Partiti (8), and Tombus (6) families (Figure 1, Figure 2 and Figures S1–S4).

**Figure 3:**
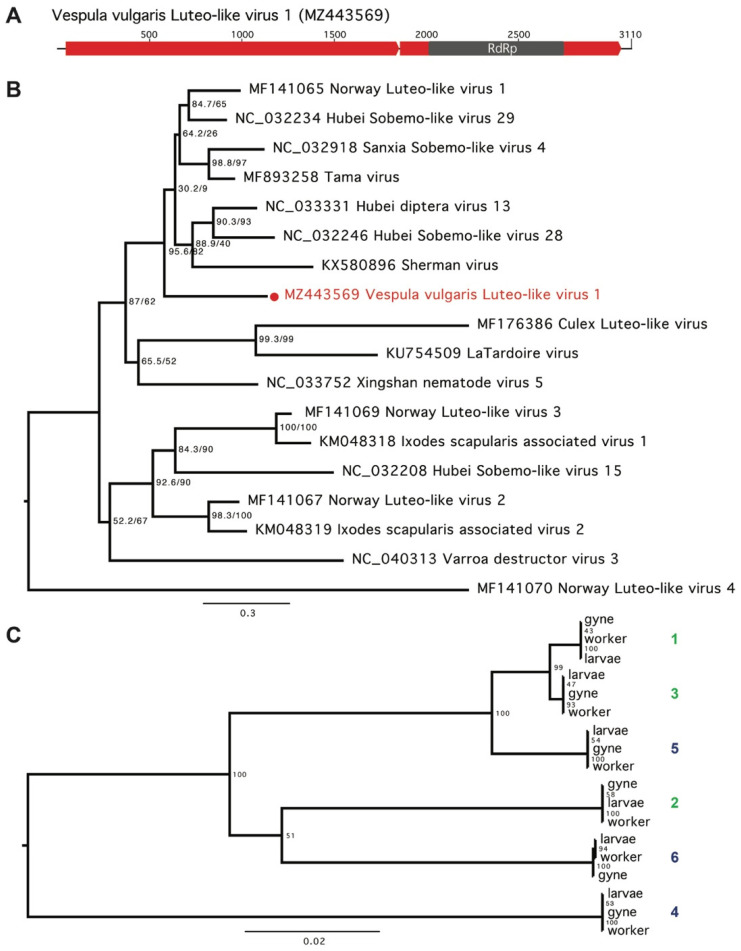
Vespula vulgaris Luteo-like virus 1. (**A**) The 3110-nucleotide genome contains two predicted ORFs (red). Conserved functional domains are indicated in grey. (**B**) Maximum likelihood phylogenetic tree of 18 selected Luteo/Sobemo viruses (GenBank accession numbers indicated), with Vespula vulgaris Luteo-like virus 1 indicated in red. After trimming, amino acid alignment length was 208 residues. The phylogenetic tree was generated in IQ-TREE, with the LG + I + G4 substitution model that had the optimal BIC score as determined by ModelFinder. Branch supports were estimated using ultrafast bootstrap approximation (UFBoot) using 1000 replicates. (**C**) Maximum likelihood phylogenetic tree comparing Vespula vulgaris Luteo-like virus 1 nucleotide sequences from all samples in six colonies (1–3: New Zealand; 4–6: Belgium). Sequences from each sample type (larvae, worker and gyne) form clades based on colony. The phylogenetic tree was generated in IQ-TREE, with the TIM2 + F + I substitution model that had the optimal BIC score. Branch supports were estimated using UFBoot with 1000 replicates.

We found one Luteo-like virus, Vespula vulgaris Luteo-like virus 1, present in all samples (Figure 2 and Figure 3, Table S4). The 3110-nucleotide, positive-sense, single-stranded RNA viral genome contains two predicted ORFs (Figure 3A) and was present at moderate abundance in all three life stages from all six colonies (0.02–0.43% of total RNA, at 46–7378-fold average coverage, Table S5). Vespula vulgaris Luteo-like virus 1 showed up to 10% nucleotide variation between samples, with variation clustered by colony (Figure 3C).

Using PCR primers designed to amplify Vespula vulgaris Luteo-like virus 1 (Table S2), we sampled four additional V. vulgaris workers from Belgium collected in 2015 and detected the virus in all four samples. We also validated the full viral genome sequence by sequencing from an independent sample. PCR primers designed from the Luteo-like virus genome amplified two long overlapping products and one shorter product to sequence a difficult region. Sanger sequences of the PCR products aligned with the assembled sequence, providing complete coverage except for a 4 bp gap at positions 2331–2334 (File S2).

**Figure 4:**
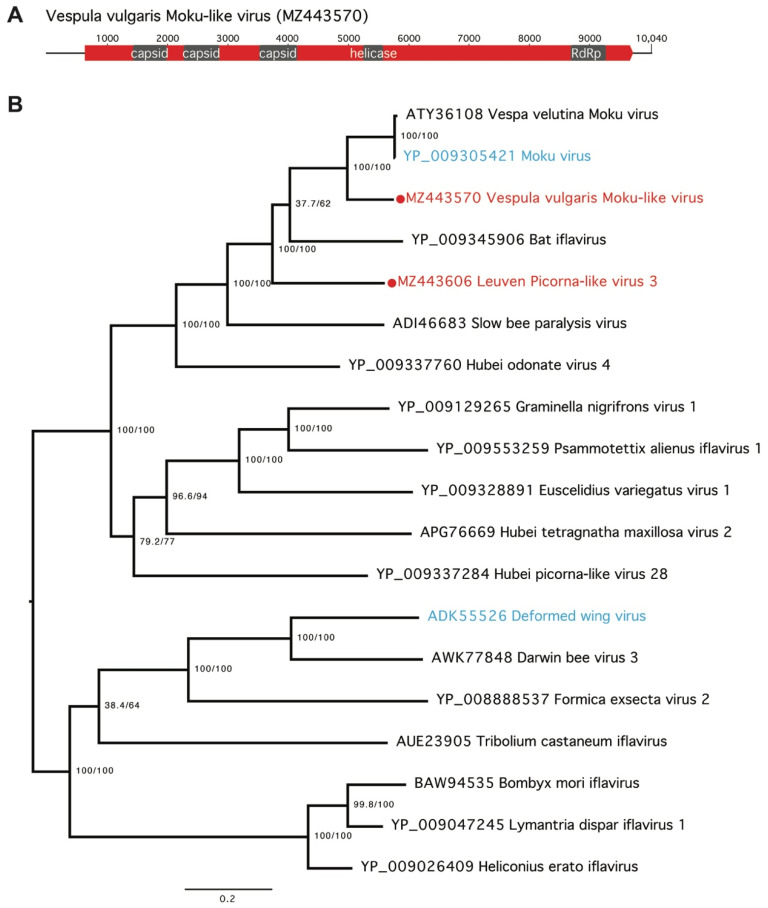
Vespula vulgaris Moku-like virus. (**A**) The 10,040-nucleotide genome contains one predicted large open reading frame (red). Conserved functional domains are indicated in grey. (**B**) Maximum likelihood phylogenetic tree of amino acid sequences of 19 selected Picorna viruses from the Iflaviridae on GenBank, with Vespula vulgaris Moku-like virus and another novel Picorna virus indicated (red). Known viruses also present in our samples are indicated in blue. The phylogenetic tree was generated in IQ-TREE, with the LG + F + I + G4 substitution model, which had the optimal BIC score as determined by ModelFinder Branch supports were estimated using ultrafast bootstrap approximation (UFBoot) using 1000 replicates.

We also identified a novel Iflavirus, Vespula vulgaris Moku-like virus (Figure 2 and Figure 4), with 71% amino-acid identity to Moku virus, originally identified in *Vespula pensylvanica* [5]. Vespula vulgaris Moku-like virus consists of a 10,040-nucleotide positive-sense, single-stranded RNA genome with a single predicted ORF (Figure 4A) and was present in two colonies from Belgium (Tables S4 and S5). Four individual *V. vulgaris* collected in Belgium in 2015 were PCR-tested for Moku-like virus with primers designed from the assembled genome. Of the four samples, two were positive using the three screening PCR primer pairs (Table S2). The full Moku-like virus genome was validated by Sanger sequencing from one of the positive samples. Nine overlapping PCR products aligned to the assembled genome with complete coverage except for a lack of consensus at positions 5820–5821 (File S2). To determine if Moku-like replication was occurring in *V. vulgaris*, we attempted to detect the presence of the negative strand produced during viral replication of positive stranded RNA viruses using strand-specific RT-PCR [48,53]. After repeated attempts, we were unable to clearly detect the Moku-like virus negative strand due to the presence of false-positives, which is one of the common difficulties encountered with strand-specific PCR [53,54].

Due to the similarity between the novel Vespula vulgaris Moku-like virus and the original Moku virus isolated in *V. pensylvanica*, we hypothesised that the novel Moku-like virus might also show cross-infectivity to honey bees. We conducted feeding experiments where we administered wasp homogenate from a Moku-like virus positive sample to adult honey bees. The homogenate prepared from the Moku-like positive wasp also tested positive for Luteo-like virus. PCR analysis indicated that 100% (17 of 17) of honey bees fed virus homogenate containing both viruses remained positive for Moku-like virus for seven days after infection, while 0% were positive for Luteo-like virus. No overt effects of the virus infections were observed on bee health or longevity over the short duration of this experiment. None of the honey bees in the control group showed evidence of infection by the Moku-like or Luteo-like viruses.

**Figure 5:**
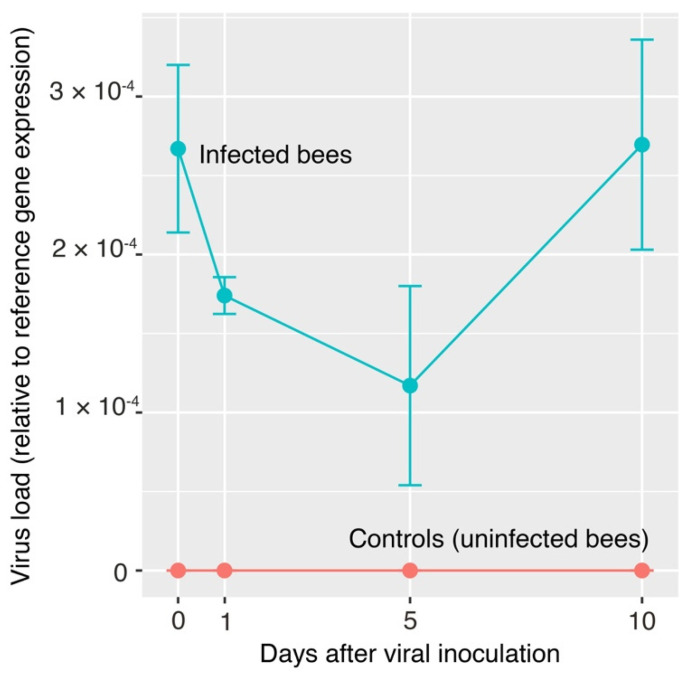
Viral load of honey bees infected with Vespula vulgaris Moku-like virus, relative to reference gene (NDUFA8 and PROS54) expression. Each viral load expression value is the average of three biological replicates. The error bars represent the standard error of the mean (SEM).

To confirm that Vespula vulgaris Moku-like viral replication was occuring in honey bees, we performed a timecourse experiment using quantitative RT-PCR to analyze viral load in bees fed Moku-like positive wasp homogenate, sampled at days 0, 1, 5, and 10. Moku-like virus was detected in bees six hours after feeding (day 0; 2.67 × 10^−4^ mean viral load), indicating viral particles had been successfully ingested into the bee gut. We then saw an initial drop in viral load in bees examined at days 1 and 5, followed by an increase at day 10. The relative level of viral infection at day 10 was 2.3-fold higher than at day 5 (Figure 5).

## 4. Discussion

We observed a highly diverse virome in *V. vulgaris* wasps, with 68 novel and seven previously identified virus genomes from 11 viral families, found in larvae, workers, and gynes from six nests sampled in two geographic locations. All nests were sampled over a single season (Autumn 2014 in both ranges) from sampling locations of sufficient distance to ensure independence (>30 km), other than two colonies from Belgium that were approximately 4 km from each other (colonies 4 and 6; Table S1). The range of different viruses observed in a relatively small sample size suggests that the underlying virus diversity in wasps is far greater than that identified here, and isolation of novel viruses is limited only by how extensive the sampling may be. However, viral presence alone is insufficient evidence to assume a newly identified virus is infectious to the sampled host. Our samples comprised total RNA taken from five whole individuals of each life stage. Therefore, it is also possible that some of the viruses that we observed could be environmentally acquired from the diet or be part of the external surface microbiota. Our results might thus represent the viruses to which these wasps are exposed, rather than the viruses that specifically infect these wasps.

There was a clear bias towards more virus sequences being present in larvae (Figure 1, Table S3). Wasps are omnivorous and have a highly polyphagous diet consisting of a diverse array of nectar, honeydew, and arthropod prey [55]. Many of the novel virus sequences are most similar to viruses previously identified in arthropods, belong to common arthropod virus families (Table S4, File S1; [1]), and may reflect the viral content of prey organisms present in the gut of feeding *Vespula* larvae. The occurrence of 17 partial viral genomes, many of which were isolated in larvae (Table S4), lends further support to this hypothesis, as the assembly of full-length viral genomes might be hindered by degradation that occurs during digestion. Viral metatranscriptomics and metagenomics can therefore inform us about the types of prey used by invasive predators such as *Vespula* wasps. Many insect viruses are multi-host pathogens [10,18,22], suggesting that wasps could be susceptible to viral infection sourced from their diet. *Vespula* species may therefore require enhanced anti-viral or other immune mechanisms to combat increased exposure to dietary pathogens.

In contrast to larvae (4–31 viruses per sample), gynes were relatively depauperate of viruses (1–3 viruses per sample), which could be due to sampling time and/or life stage. Wasp larvae hang in their nest cells by their abdomen, head down. They retain digestive waste products within their gut and defecate only when the larvae pupate [56], likely only then purging some of the viruses that they have obtained from the diet. Furthermore, the gynes we sampled in autumn are likely to be newly emerged adults that have had a relatively short time or few opportunities to become re-infected with pathogens. Workers, in contrast, have an adult foraging tenure of up to 43 days, consisting of over 800 foraging trips [57]. These workers (4–9 viruses per sample) would have had the opportunity to be exposed to a wide variety of pathogens while foraging. Notably, the three viruses present in gynes were Vespula vulgaris Luteo-like virus 1, Vespula vulgaris Moku-like virus, and Moku virus, which could also indicate a higher host-infectivity of these viruses allowing them to more easily infiltrate a colony, including newly emerged gynes.

Other measures such as abundance or prevalence can be crude indicators that a virus genuinely infects a host. For example, Vespula vulgaris Luteo-like virus 1 was isolated in all 18 samples (six colonies, three life stages) from both the native and invasive range at relatively high abundance (ranging from 50- to 7000-fold coverage), which is a strong indicator that this virus is a genuine *Vespula*-infecting species. Luteo-Sobemo viruses are common plant pathogens, however a related but diverged group of Luteo-like viruses have recently been identified in a range of arthropods including mosquitoes and ticks (Figure 3B) [1,58,59]. The repeated identification of a virus sequence in a particular species is a strong indication that a virus genuinely infects that host. We saw 13 viruses (nine novel) present in more than one colony, and four present in both the native and invasive range (Luteo-like virus 1, Sobemo-like virus 1, Permutotera-like 1 and Deformed wing virus), suggesting these viruses are perhaps more likely to be genuine wasp-infecting viruses.

Our data support previous observations that pollinator viromes are dominated by picornaviruses [6], with 30 picorna virus genomes (of which 24 were novel) being present in our samples. In addition, our characterization of a second virus closely related to Moku virus from *V. pensylvanica* suggests that there is an emerging clade of Moku-like viruses present in *Vespula* species (Figure 4B) [5]. We found Vespula vulgaris Moku-like virus in multiple wasp colonies in Belgium across two independent seasons (2014 and 2015). We selected Moku-like virus for further study in honey bees because of the close similarity to Moku-virus, a known multi-host pathogen. Indeed, phylogenetically related viruses tend to behave similarly in terms of host preference, such that evolutionary relatedness may be a good predictor of novel spillover events [60]. Our feeding experiments indicate that Vespula vulgaris Moku-like virus has potential cross-infectivity with honey bees, but that Vespula vulgaris Luteo-like virus 1 does not. The cross-infectivity of Vespula vulgaris Moku-like virus is perhaps not surprising given previous observations of the original Moku virus having been found in the wasps *Vespula pensylvanica* and *V. vulgaris*, honey bees, Argentine ants, other ants, mites parasitizing wasps, and even spiders [20,38,53]. Moku-like virus loads persisted in honey bee workers over time after infection via feeding (Figure 5). Shortly after feeding, viral loads were initially high, which most likely represents the viral dose administered. Then, we observed a drop in viral load between days 1–5, likely due to the passage of ingested virus through the digestive tract, followed by an increase in viral load at day 10, suggesting that an active infection had become established. Our infection assay does not demonstrate viral replication in bees per se, although due to the infection profile we assume that it must have been occurring. Using similar assays, we have previously confirmed viral replication in wasps and spiders [18,20,23].

It will be important to determine the prevalence and pathogenicity of the novel Moku-like virus in global wasp and honey bee populations, given that the closely related Moku virus has now been widely isolated [35,36,37] and has potential fitness impacts on hosts [33]. Control of *Vespula* wasp populations may be achievable through the discovery of novel viruses that infect them [61], however, consideration of potential spillover effects into commercial pollinators is critical given that pathogen sharing is widespread amongst Hymenoptera [25].

## 5. Conclusions

The invasive common wasp, *Vespula vulgaris*, harbors a complex virome, as demonstrated by the 68 novel and seven previously identified virus genomes from 11 viral families described here. These viruses are likely to be shared among arthropod species, including honey bees that underpin pollination and primary industries in many countries. The invasion of wasp populations into a new range brings an obvious direct predation pressure, but also can bring a hidden and very diverse package of pathogens that may influence the population dynamics of the wider species pool.

## Data Availability

Raw FastQ files for each transcriptome sample have been submitted to NCBI Sequence Read Archive under the Bioproject PRJNA740346, with sample accession numbers SAMN19842383–SAMN19842400. Novel virus sequences identified in this study have been submitted to GenBank (GB) with the GB identifiers MZ443569–MZ443636.

## Supplementary Materials

The following are available online at https://www.mdpi.com/article/10.3390/v13081431/s1, Table S1: Colony site coordinates and date of collection for *V. vulgaris* samples used for total RNA transcriptomes from the native Belgian range and invasive New Zealand range. Table S2: Primers used in this study. Table S3: Total number of viruses present, and number of virus-like contigs assembled de novo from each transcriptome. Table S4: Novel virus sequences identified in transcriptomes of *Vespula vulgaris* larvae, workers, and gynes in colonies from Nelson, New Zealand and Leuven, Belgium. Table S5: Average fold coverage and percentage of RNA reads aligning to Vespula vulgaris Luteo-like virus 1 and Vespula vulgaris Moku-like virus. Figure S1: Detailed Picorna phylogenetic tree from Figure 1. Figure S2: Detailed Luteo/Sobemo phylogenetic tree from Figure 1. Figure S3: Detailed Partiti phylogenetic tree from Figure 1. Figure S4: Detailed Tombus phylogenetic tree from Figure 1. File S1: Summary of viruses identified. File S2: Sanger sequences confirming Vespula vulgaris Luteo-like virus 1 and Vespula vulgaris Moku-like virus genomes.
