# Supplementary material for: A Diverse Viral Community from Predatory Wasps in Their Native and Invaded Range, with a New Virus Infectious to Honey Bees
Source: Viruses. 2021 Jul 23;13(8):1431. doi: 10.3390/v13081431 (PMC8402789; doi:10.3390/v13081431)
Supplement: Supplementary file 1 [file viruses-13-01431-s001.zip › viruses-1304531-supplementary/Supplementary file submission/Supplementary Tables and Figures.pdf]

Table S1. Colony site coordinates and date of collection for *V. vulgaris* samples used for total RNA transcriptomes from the native Belgian range and invasive New Zealand range.

Range	Colony	Site	Coordinates	Date
**Invasive** New Zealand	1	Six Mile Reserve	S -41.7685; E 172.9572	March 2014
	2	Tin Line Reserve	S -41.2809; E173.5087	March 2014
	3	Braeburn Track	S -41.7954; E 172.5156	March 2014
**Native** Belgium	4	Wijgmaal, Leuven	N 50.5539; E 4.4143	September 2014
	5	Hasselt, Hasselt	N 50.5523; E 5.2017	September 2014
	6	Wezemaal, Leuven	N 50.5720; E 4.4444	September 2014

Table S2. Primers used in this study. *indicates Moku-like virus primers that were used for qPCR.

Name	Sequence (5′-3′)	Product size (bp)	
Virus primers		*Sequencing*	*Screening*
Moku-like_88F	GTAACTGGCTATCTCTGCTCG	1195	
Moku-like_1282R	GGTAACATCTCAATATACACACCC		
Moku-like_1119F	GTTGATACGATTGTTAATATTTGGG	1093	
Moku-like_2211R	TAATTGGTTCTCTAGTCTTAGTTCC		106
Moku-like_2106F	GGAGATGTTAATTGTGATAATCCC	1391	
Moku-like_3496R	GGAGTCACATACGTCTCTTCC		279
Moku-like_3218F	TGTTGGATGTGCTATTAGTTGG	1128	
Moku-like_4345R	TTGCCAAAGAGAGTATTCATTCC		
Moku-like_4265F	CATCCCTGTAGTGTACGC	1118	
Moku-like_5382R	GCCAGTATATCATACATTCTTGG		
Moku-like_5299F	GGAAGTCACATATAACTCAGC	1307	
Moku-like_6605R	GACTTTATCCAATGTATCACCAC		
Moku-like_6511F	CAATTCGATCTTATGTTTACTGG	1120	
Moku-like_7630R	GTTAAAGGTTTAGTAAGCATTTCC		
Moku-like_7517F	GAATGATGATGATGGAAATGCG	1290	
Moku-like_8806R*	CCAGGAATAGCACATTTCGC		143
Moku-like_8664F*	CGTCTTAAAGGCTTAGCACC	1255	
Moku-like_9918R	CCGTGTTATAGCCACAACG		
Luteo-like_11F	GCGTTTCCGTTTCTATATTTTTGTG	1685	
Luteo-like_1695R	GACGATTTCTTCTGCTTCTTCG		372
Luteo-like_1324F	GGACCAACTTATTCAGACTGC	1790	
Luteo-like_3113R	CACCTTTGTTTCACCTCACAC		
Luteo-like_2364F	AAGAGTTGGCTGGTCCCCTT	483	
Luteo-like_2846R	CCCTTGAAGAGCATACCGGC		
Reference gene primers for qPCR [1]			
Ndufa8_Apis_F	GCACGATTCACCAAGACCAA		78
Ndufa8_Apis_R	GGTGGAGCTACAGGCTCAGG		
Pros54_Apis_F	TCGAACCAAGATGGTACTGGAA		100
Pros54_Apis_R	TTGTTGTGCTTGCAGTCGTG		

Table S3. Total number of viruses present, and number of virus-like contigs assembled *de novo* from each transcriptome. The percentage of RNA-seq reads aligning to virus in each sample is indicated. (Colonies from Nelson, New Zealand (1-3) and Leuven, Belgium (4-6).

		Larvae						Workers						Gynes					
		**1**	**2**	**3**	**4**	**5**	**6**	**1**	**2**	**3**	**4**	**5**	**6**	**1**	**2**	**3**	**4**	**5**	**6**
Total viruses present		7	11	4	7	21	31	9	5	4	7	5	4	1	2	1	1	3	1
# virus-like contigs	*>200 bp*	105	243	54	102	337	403	45	9	16	90	114	71	2	3	1	1	5	2
	>1000 bp	15	48	8	12	50	69	13	5	8	23	15	8	2	2	1	1	3	1
% of reads aligned to virus		0.27	0.15	0.08	0.6	0.75	0.76	0.21	0.43	0.05	0.99	0.49	0.27	0.04	0.22	0.05	0.13	0.15	0.17

Table S4. Novel virus sequences identified in transcriptomes of *Vespula vulgaris* larvae, workers and gynes in colonies from Nelson, New Zealand (1-3) and Leuven, Belgium (4-6). Presence in multiple samples is indicated by shaded box. Viruses 1-9 (bold) were identified in more than one colony so have been named 'Vespula vulgaris [virus]'. Other viruses identified in one colony or sample only are named based on location. If a clear viral family is evident based on homology, viruses have been named according to family (eg. Nelson Picorna-like virus 1), otherwise, for unclassified or uncharacterised viral families 'wasp-associated virus' has been used. Supplementary file 1 contains further details including virus sequences. (Table continued on following page).

		GB accession	Closest match (GB)	Similarity (AA)	Size (bp)	Full genome	Larvae						Workers						Gynes					
							1	2	3	4	5	6	1	2	3	4	5	6	1	2	3	4	5	6
1	***Vespula vulgaris* luteo-like virus 1**	MZ443569	MF141065	45%	3110	Likely	■	■	■	■	■	■	■	■	■	■	■	■	■	■	■	■	■	■
2	***Vespula vulgaris* Moku-like virus**	MZ443570	NC_031338	71%	10040	Likely					■	■					■						■	
3	***Vespula vulgaris* Sobemo-like virus 1**	MZ443571	NC_033138	79%	3141	Likely				■			■											
4	***Vespula vulgaris* Permutotera-like virus 1**	MZ443572	NC_033114	44%	4862	Partial					■		■			■		■						
5	***Vespula vulgaris* Picorna-like virus 1**	MZ443573	NC_003924	48%	9290	Likely	■	■																
6	***Vespula vulgaris* Partiti-like virus 1**	MZ443574	KX884166	65%	1442	Segment	■	■																
7	***Vespula vulgaris* Partiti-like virus 2**	MZ443575	KX884140	54%	1691	Segment	■	■																
8	***Vespula vulgaris* Luteo-like virus 2**	MZ443576	MH188016	45%	2714	Likely				■	■	■					■							
9	***Vespula vulgaris* Picorna-like virus 2**	MZ443577	QNS17456	79%	9145	Likely				■		■				■		■						
10	Nelson Picorna-like virus 1	MZ443578	NC_035450	55%	8353	Partial	■																	
11	Nelson Picorna-like virus 2	MZ443579	NC_033126	42%	8629	Likely	■																	
12	Nelson Picorna-like virus 3	MZ443580	NC_033173	72%	9536	Likely		■																
13	Nelson Picorna-like virus 4	MZ443581	NC_030843	56%	8285	Likely		■																
14	Nelson Picorna-like virus 5	MZ443582	KY316058	66%	6729	Partial		■																
15	Nelson Picorna-like virus 6	MZ443583	NC_033437	50%	10071	Likely		■																
16	Nelson Picorna-like virus 7	MZ443584	NC_033437	56%	9578	Likely		■																
17	Nelson Picorna-like virus 8	MZ443585	NC_035450	57%	11865	Likely		■																
18	Nelson wasp-associated virus 1	MZ443586	KX580890	60%	5281	Partial		■																
19	Nelson wasp-associated virus 2	MZ443587	KX235518	44%	2314	Likely			■						■									
20	Leuven wasp-associated virus 1	MZ443588	NC_033491	80%	3730	Likely				■						■								
21	Leuven Picorna-like virus 1	MZ443589	NC_002066	36%	9022	Likely				■														
22	Leuven Picorna-like virus 2	MZ443590	KY969634	46%	10231	Likely					■													
23	Leuven Virga-like virus 1	MZ443591	NC_033192	43%	7907	Partial					■													
24	Leuven wasp-associated virus 2	MZ443592	NC_033425	23%	10373	Likely					■													
25	Leuven wasp-associated virus 3	MZ443593	NC_033462	48%	8983	Likely					■													
26	Leuven Sobemo-like virus 1	MZ443594	NC_033138	43%	2806	Likely					■													
27	Leuven Sobemo-like virus 2	MZ443595	NC_032233	51%	2707	Likely					■													
28	Leuven Nodavirus-like 1	MZ443596	NC_032872	52%	2848	Likely					■													
29	Leuven Nodavirus-like 2	MZ443597	NC_002690	44%	3190	Likely					■													
30	Leuven Tombus-like virus 1	MZ443598	NC_032455	53%	2203	Likely					■													
31	Leuven Bunya-like virus 1	MZ443599	MH213237	24%	1861	Partial					■													
32	Leuven Tetra-like virus 1	MZ443600	AF062037	37%	1858	Partial					■													
33	Leuven wasp-associated virus 4	MZ443601	NC_033709	35%	4200	Likely					■													
34	Leuven wasp-associated virus 5	MZ443602	KX883912	61%	9599	Likely						■												
35	Leuven wasp-associated virus 6	MZ443603	NC_033709	35%	5704	Likely						■												
36	Leuven Sobemo-like virus 3	MZ443604	NC_032937	72%	3182	Likely						■												
37	Leuven Luteo-like virus 1	MZ443605	MF176307	43%	2971	Likely						■												
38	Leuven Picorna-like virus 3	MZ443606	NC_033823	54%	10442	Likely						■												
39	Leuven Picorna-like virus 4	MZ443607	NC_032756	49%	5723	Partial						■												
40	Leuven Picorna-like virus 5	MZ443608	NC_033173	47%	9773	Likely						■												
41	Leuven Picorna-like virus 6	MZ443609	NC_033173	51%	7016	Partial						■												
42	Leuven Picorna-like virus 7	MZ443610	MG995701	41%	6198	Partial						■												
43	Leuven Picorna-like virus 8	MZ443611	MG995697	36%	5530	Partial						■												
44	Leuven Picorna-like virus 9	MZ443612	NC_033040	46%	6705	Partial						■												
45	Leuven Picorna-like virus 10	MZ443613	NC_032221	30%	8321	Likely						■												
46	Leuven Picorna-like virus 11	MZ443614	NC_032993	63%	5120	Partial						■												
47	Leuven Picorna-like virus 12	MZ443615	NC_033714	37%	11161	Likely						■												
48	Leuven Tombus-like virus 2	MZ443616	KX883118	50%	2767	Likely						■												
49	Leuven Tombus-like virus 3	MZ443617	NC_032453	34%	3758	Likely						■												
50	Leuven Tombus-like virus 4	MZ443618	NC_033017	69%	3907	Partial						■												
51	Leuven Tombus-like virus 5	MZ443619	NC_032970	73%	4790	Likely						■												
52	Leuven Tombus-like virus 6	MZ443620	MG008317	48%	3408	Likely						■												
53	Leuven Nodavirus-like 3	MZ443621	NC_014978	45%	3803	Likely						■												
54	Leuven Partiti-like virus 1	MZ443622	MF346350	55%	1506	Segment						■												
55	Leuven Partiti-like virus 2	MZ443623	KX884166	57%	1463	Segment						■												
56	Leuven Partiti-like virus 3	MZ443624	NC_032139	55%	1693	Segment						■												
57	Leuven Partiti-like virus 4	MZ443625	KX884105	53%	1400	Segment						■												
58	Nelson Astrovirus-like 1	MZ443626	MF042208	76%	5832	Likely							■											
59	Nelson wasp-associated virus 3	MZ443627	NC_033139	92%	3095	Likely							■											
60	Nelson Nodavirus-like 1	MZ443628	NC_002690	49%	3124	Likely							■											
61	Nelson Picorna-like virus 9	MZ443629	NC_033194	51%	7829	Partial							■											
62	Nelson Sobemo-like virus 1	MZ443630	NC_033138	52%	3037	Likely								■						■				
63	Nelson Narna-like virus 1	MZ443631	NC_033013	66%	3110	Likely								■										
64	Nelson Partiti-like virus 1	MZ443632	MF346350	52%	1394	Segment								■										
65	Nelson Bunya-like virus 1	MZ443633	NC_032282	44%	7698	Segment									■									
66	Leuven Bunya-like virus 2	MZ443634	NC_031293	30%	6338	Partial										■								
67	Leuven wasp-associated virus 7	MZ443635	NC_032925	36%	3727	Partial										■								
68	Leuven Partiti-like virus 5	MZ443636	KX884140	72%	1689	Segment											■							
	**Previously identified virus sequences**						1	2	3	4	5	6	1	2	3	4	5	6	1	2	3	4	5	6
1	Kashmir bee virus	NC_004807					■		■				■	■	■									
2	Deformed wing virus	NC_004830							■			■												
3	Rhopalosiphum padi virus	NC_001874								■	■					■								
4	Moku virus	NC_031338									■						■							
5	Riboviria sp. isolate cfe153shi01	MT138203									■	■						■						
6	Aphid lethal paralysis virus	NC_004365										■												
7	Prestney Burn virus	KU754507									■													
8	Sanxia water strider virus 16	NC_033139											■											
9	Ribgrass mosaic virus	NC_002792									■													

Table S5: Average fold coverage and percentage of RNA reads aligning to *Vespula vulgaris* Luteo-like virus 1 and *Vespula vulgaris* Moku-like virus. (Colonies from Nelson, New Zealand (1-3) and Leuven, Belgium (4-6)).

		**Larvae**						**Workers**						**Gynes**					
		**1**	**2**	**3**	**4**	**5**	**6**	**1**	**2**	**3**	**4**	**5**	**6**	**1**	**2**	**3**	**4**	**5**	**6**
Luteo-like virus	Fold coverage	1630	286	841	5030	46	7378	969	974	827	2964	2391	4006	480	519	550	1923	1026	2446
	% reads	0.12	0.02	0.08	0.34	0.01	0.43	0.06	0.07	0.04	0.15	0.22	0.23	0.04	0.04	0.05	0.02	0.06	0.17
Moku-like virus	Fold coverage					770	30					119						537	
	% reads					0.09	0.01					0.04						0.10	

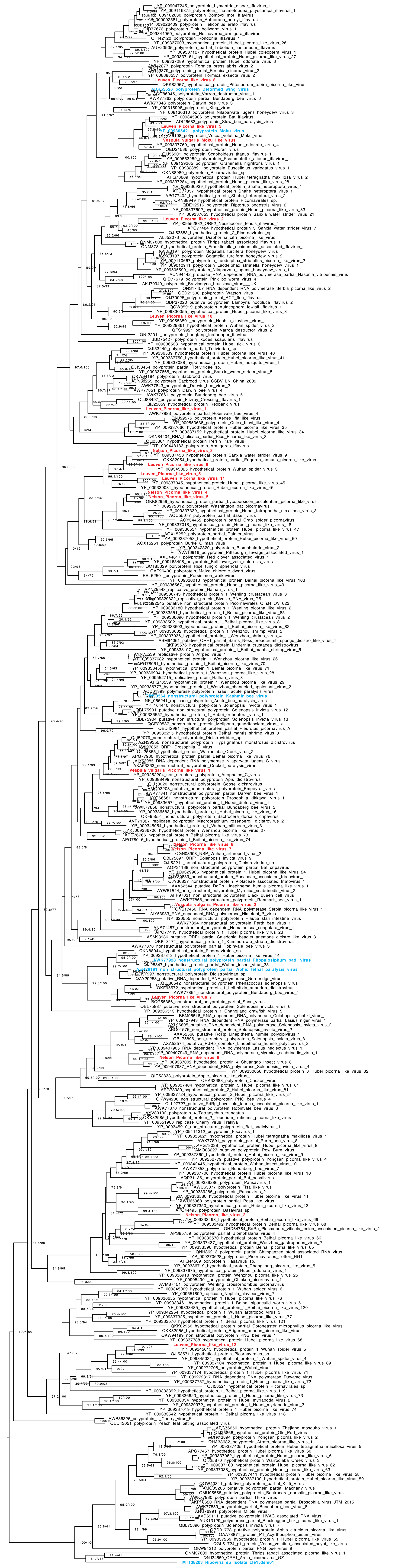

Figure S1. Detailed Picorna phylogenetic tree from Figure 1. Phylogenetic tree of the novel Picorna-like viruses identified in *Vespula vulgaris*. Novel viruses are shown in red, and previously known viruses also present in these samples are indicated in blue. A region of the polypeptide containing the RNA dependent RNA polymerase (RdRp) domain was aligned with previously characterised Picorna viruses obtained from NCBI. After trimming, alignment length was 256 aa, with a total of 337 sequences. The phylogenetic tree was generated using maximum likelihood in IQ-TREE [2], with the LG+F+I+G4 substitution model which had the optimal BIC score, as determined by ModelFinder [3]. Branch supports were estimated using Ultrafast bootstrap approximation (UFBoot; [4]) using 1000 replicates. Support values shown are SH-aLRT and UFBoot supports.

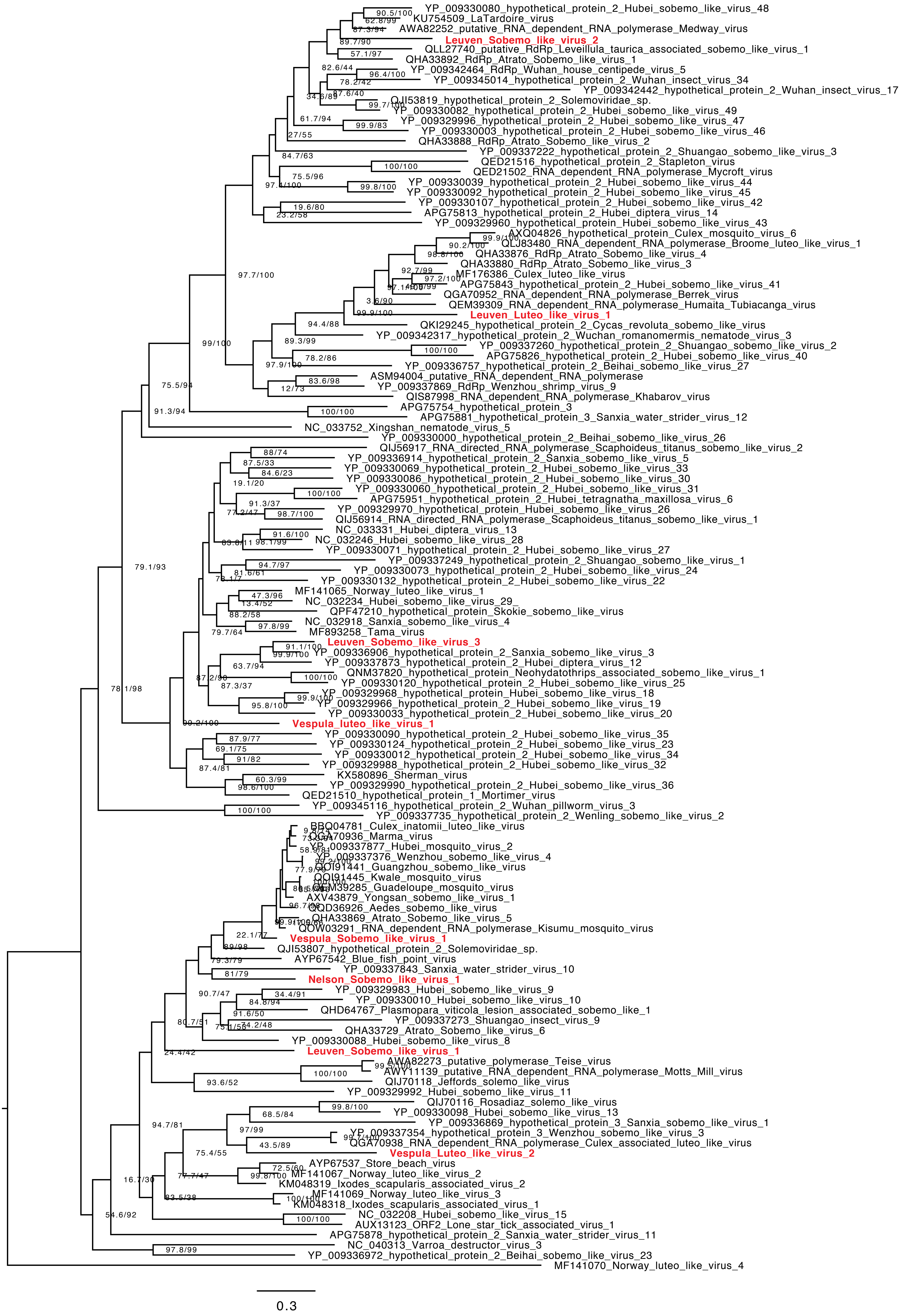

Figure S2. Detailed Luteo/Sobemo phylogenetic tree from Figure 1. Phylogenetic tree of the novel Luteo/Sobemo viruses identified in *Vespula vulgaris*. Novel viruses are shown in red. The open reading frame containing the RNA dependent RNA polymerase (RdRp) domain was aligned with previously characterised Luteo/Sobemo viruses obtained from NCBI. After trimming, alignment length was 259 aa, with a total of 124 sequences. The phylogenetic tree was generated using maximum likelihood in IQ-TREE [2], with the LG+I+G4 substitution model which had the optimal BIC score, as determined by ModelFinder [3]. Branch supports were estimated using Ultrafast bootstrap approximation (UFBoot; [4]) using 1000 replicates. Support values shown are SH-aLRT and UFBoot supports.

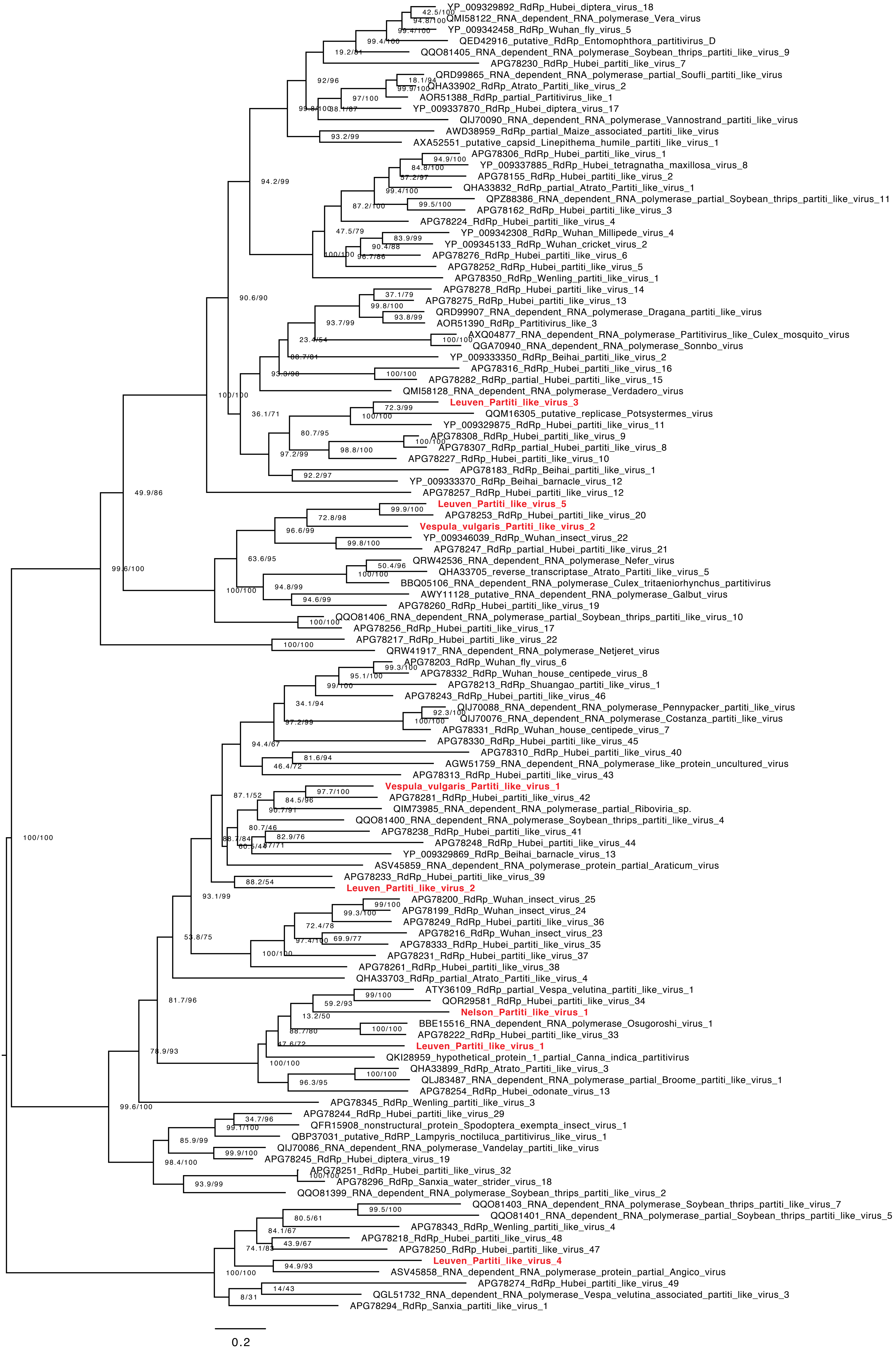

Figure S3. Detailed Partiti phylogenetic tree from Figure 1. Phylogenetic tree of the novel Partiti viruses identified in *Vespula vulgaris*. Novel viruses are shown in red. The open reading frame containing the RNA dependent RNA polymerase (RdRp) domain was aligned with previously characterised Partiti viruses obtained from NCBI. After trimming, alignment length was 405 aa, with a total of 116 sequences. The phylogenetic tree was generated using maximum likelihood in IQ-TREE [2], with the LG+F+I+G4 substitution model which had the optimal BIC score, as determined by ModelFinder [3]. Branch supports were estimated using Ultrafast bootstrap approximation (UFBoot; [4]) using 1000 replicates. Support values shown are SH-aLRT and UFBoot supports.

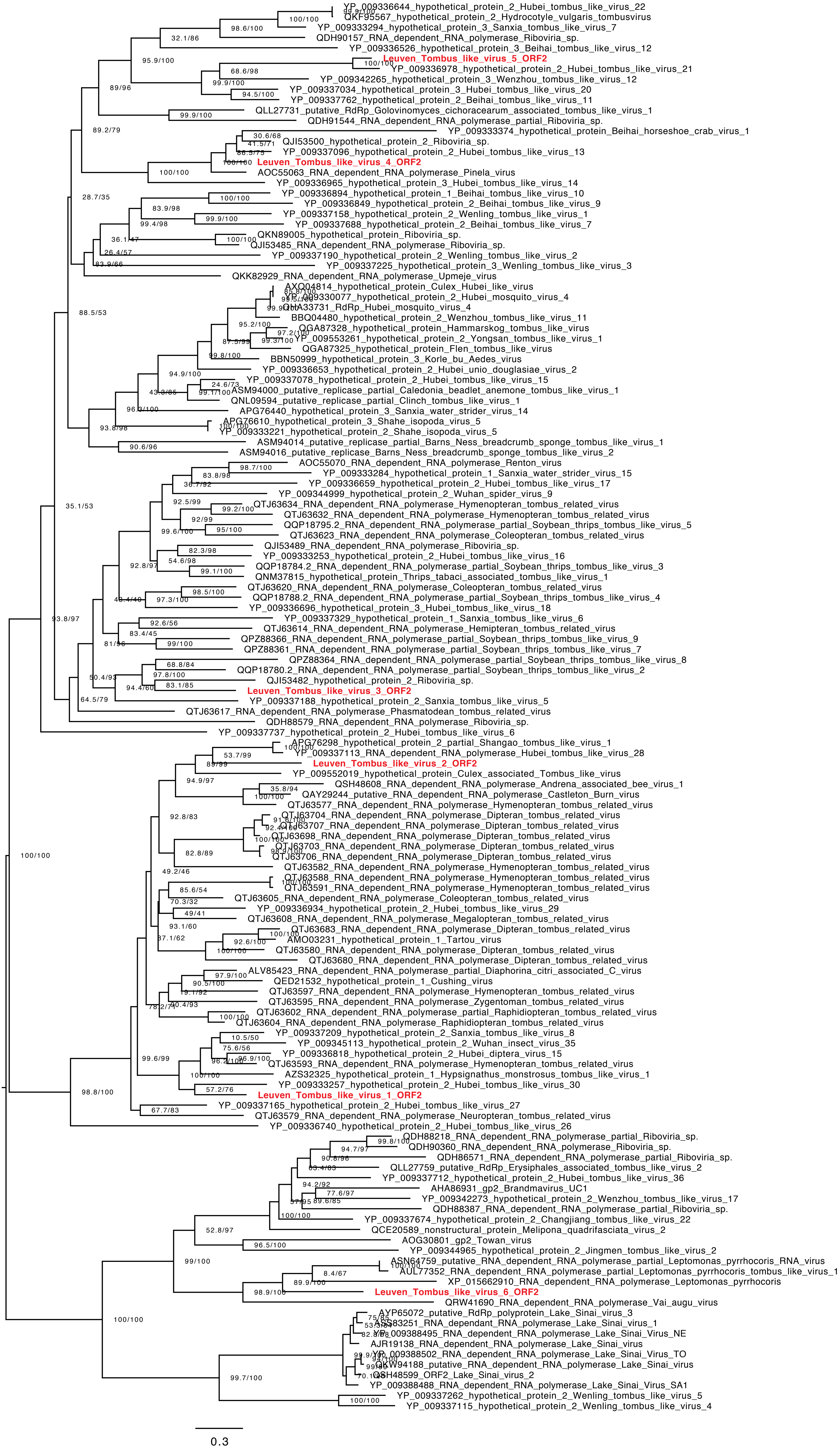

Figure S4. Detailed Tombus phylogenetic tree from Figure 1. Phylogenetic tree of the novel Tombus viruses identified in *Vespula vulgaris.* Novel viruses are shown in red. The open reading frame containing the RNA dependent RNA polymerase (RdRp) domain was aligned with previously characterised Partiti viruses obtained from NCBI. After trimming, alignment length was 344 aa, with a total of 136 sequences. The phylogenetic tree was generated using maximum likelihood in IQ-TREE [2], with the rtREV+F+I+G4 substitution model which had the optimal BIC score, as determined by ModelFinder [3]. Branch supports were estimated using Ultrafast bootstrap approximation (UFBoot; [4]) using 1000 replicates. Support values shown are SH-aLRT and UFBoot supports.

Supplementary references

1. Cameron, R.C.; Duncan, E.J.; Dearden, P.K. Stable reference genes for the measurement of transcript abundance during larval caste development in the honeybee. *Apidologie* **2013**, *44*, 357-366.
2. Nguyen, L.-T.; Schmidt, H.A.; von Haeseler, A.; Minh, B.Q. Iq-tree: A fast and effective stochastic algorithm for estimating maximum-likelihood phylogenies. *Mol Biol Evol* **2014**, *32*, 268-274.
3. Kalyaanamoorthy, S.; Minh, B.Q.; Wong, T.K.F.; von Haeseler, A.; Jermiin, L.S. Modelfinder: Fast model selection for accurate phylogenetic estimates. *Nat Methods* **2017**, *14*.
4. Hoang, D.T.; Chernomor, O.; von Haeseler, A.; Minh, B.Q.; Vinh, L.S. Ufboot2: Improving the ultrafast bootstrap approximation. *Mol Biol Evol* **2017**, *35*, 518-522.
